# Encouraging a Little Help from Our Friends: Resident Physician Burnout & Peer Communication Curriculum

**DOI:** 10.51894/001c.22044

**Published:** 2021-04-13

**Authors:** Brenda Lovegrove Lepisto

**Affiliations:** 1 Family Medicine, McLaren Greater Lansing; College of Osteopathic Medicine, Michigan State University

**Keywords:** mental health stigma, curriculum, roleplaying, residents, burnout

## Abstract

**INTRODUCTION:**

Resident physician burnout and depression rates are increasing faster than in the non-physician workforce. To foster a supportive community where such concerns may be addressed, residents can be educated in identification and first-line support of burnout in fellow residents. The literature has not described peer roleplaying applied toward aiding fellow residents with burnout.

**METHOD:**

This pilot study evaluated an educational component aimed at fostering a strong emotional and informational social support system. The curriculum used peer roleplaying to develop self-awareness and social support, improve communication skills, and teach about existing mental health resources, thereby encouraging intervention. Residents listed behavioral manifestations of burnout and dysphoria that we developed into real-life scenarios. During experiential workshops, residents roleplayed “distressed” and “helper” residents and practiced communicating empathy. To tackle mental health stigma, all were required to practice expressing distress and seeking help. Residents completed a pre-roleplaying questionnaire, curriculum satisfaction questionnaire, and reflection essay.

**RESULTS:**

All 42 Internal Medicine and Transitional Year residents (69% male, 93% international medical graduates) participated. Resident-reported comfort, competence, confidence, and knowledge increased, as did positive appraisals of the clinical teaching environment representing a safe atmosphere. Six themes were cited in >25% of essays: knowledge of communication techniques, knowledge of approach tactics or strategies, knowledge of hospital resources, commitment to helping colleagues, importance of burnout, and belief this training produced a better understanding of oneself.

**CONCLUSION:**

As first-witnesses of resident physician distress, peers occupy an underutilized, yet crucial preventive and supportive role in burnout and mental health intervention, especially during times of shared crises such as the coronavirus pandemic. Creating roleplays from personal experiences facilitated meaningful discussion of burnout and dysphoric emotions. Roleplaying offered a low-cost, effective method to destigmatize and encourage discussion of burnout, educate on signs and symptoms, and learn available resources to offer an afflicted colleague in osteopathic and allopathic residency programs.

## INTRODUCTION

With resident physician burnout and depression rates increasing faster than in the non-physician workforce,^1^ the Accreditation Council for Graduate Medical Education (ACGME) now includes well-being program requirements for allopathic and osteopathic resident physicians.^2^ However, limited resources for identification and intervention of burnout exist in many allopathic and osteopathic medical resident education settings.^3–5^

Improved communication skills lead to higher physician satisfaction and lower burnout rates.^6^ Despite the common notion that simulated patient communication training yields better results than role-playing communication scenarios, a systematic review comparing the two methods found no significant differences between these two communication educational methods.^7^ Peer role-play fosters more empathy and may have added benefits with less cost than patient actors.^8^ Although peer role-playing improves communication skills,^9^ the application of this approach toward aiding fellow residents with burnout has been rarely described in the literature.

One 2019 study utilized an OSCE to train residents to identify and intervene when a distressed role-playing resident gave a colleague a sign-out report.^10^ A similar 2018 workshop used reverse role-playing to increase cognitive flexibility that has been purported to increase wellness.^11^ Reverse role-playing, a psychotherapy technique, focuses on building insight and cueing the participants to develop alternative perspectives of peers' stressful medical education experiences rather than simulating their own perspectives. Role-playing with standardized actors has also been shown to improve communication skills, reducing burnout, although more costly than peer-role-playing.^8^

In addition, peer role-playing occurs in a setting where peers interact to foster community building^12^ as learners to adopt multiple roles, enabling a greater understanding of the experience of a distressed colleague seeking support.^7,9,11,13,14^ Further, stigma related to mental health diagnosis and treatment and concerns about license reporting questions interfere with physicians discussing emotions, distress, and symptoms.^15^ Thus, physicians resist seeking mental health treatment when distressed.^16–19^

In 2012, Jean E. Wallace, Ph.D., a sociologist who has extensively researched mental health, physician well-being, support systems, and stigma, offered insight into the sociological basis of the development of stigma and ways to reduce mental health stigma in the medical profession. Deeply rooted in medical culture and cultivated by society lies the belief that physicians possess infallibility, selflessness, and infinite resilience.^16,18,19^

Wallace proposed that the medical profession reinforces societal views of mental illness stigma by three significant factors, including the culture of medicine, collegial attitudes and communication, and health care systems' response to physicians suffering from substance abuse and mental health conditions.^16^ When physicians suffer from mental illness or substance abuse, the conspiracy of silence and shame within medicine adds a layer of stigma over the reputation of the affected physician.^16,19^

Finally, in a brief 2018 article "Combatting Clinician Burnout with Community-Building" in the *New England Journal of Medicine Catalyst* researchers identify several health systems that employ a form of community building. They conclude that as physicians attempt to lift the stigma of mental illness among patients, they also need to lift mental illness stigma from their own ranks.^12^ Community building can occur with trained facilitators conducting discussion groups where residents identify stress and coping mechanisms.^20^

### Purpose of Study

The author (i.e., a trained communication facilitator and faculty member) and study team (see Acknowledgments section) hypothesized that using the peer/social support role-playing curriculum they had created would serve as an underutilized low-cost strategy of educating residents in improved peer communication and relationship skills. Even though the study team delivered this curriculum pilot study prior to the 2019 coronavirus pandemic, peer support continues to occupy a critical role in well-being during such shared crises.^3,10,12^

## METHODS

An earlier 2017 survey of burnout and depression, utilizing the *Maslach Burnout* *Inventory*^21^ and *Patient Health Questionnaire*, *Version 9* (PHQ-9),^22^ respectively at Hurley Medical Center, a 443-bed public, nonprofit safety-net teaching hospital located in Flint, Michigan, revealed similar and alarming physician burnout rates as found in national burnout surveys.^23^ For this later study, the author’s institutional review board had approved the curriculum project design as exempt from full review during the 2016-2017 academic year. All resident data were de-identified by a research coordinator (NL in Acknowledgements) on the study team.

As a critical graduate medical education initiative, our team invited our full complement of Internal Medicine and Transitional Year residents (i.e., 42 resident physicians: 36 Internal Medicine, six Transitional Year) to participate. These combined programs were composed of 39 (93%) international medical graduates, and 29 (69%) residents identified themselves as male.

### Study Intervention

The study team members first delivered a series of one-hour workshops including a didactic presentation concerning the definition, prevalence, and incidence of burnout and depression. During this didactic period, participating residents were asked to list signs, symptoms, and emotional and behavioral manifestations that they considered indicative of burnout and dysphoric emotions in colleagues and themselves. During the workshops, residents who were unable to attend parts of the hour were provided with a brief orientation to the factors contributing to burnout and the training's purpose. The workshop refresher served all residents an additional opportunity to describe emotions, situations, and burnout manifestations on a flipchart. (Table 1).

**Table 1.** Sample Characteristics and Resident-Generated Roleplay Categories

Ages	25–35
Sex	Male/Female
Problem	Burnout, Depression, Stress, Anxiety
Situation	Too many admissions Not enough time off Travel ban Awaiting job/fellowship decision Difficult interactions with attending, colleagues, patients Commute time Medical issues Family demands/lifestyle Cultural pressure Professional development/study time
Emotions	Fear, sadness, anger, anxiety, frustration, dread
Manifestation of problems	Crying, shouting, temper, mistakes, apathy, sad face/posture, appearing down, withdrawal, hyper/hypo sleeping, isolation, increased illness, lack of self-care/less exercise, irritability, substance abuse, complaints, distraction/inability to focus, tardiness/absence, forgetfulness

The author had created three scenarios of residents expressing dysphoric emotions related to burnout, exhaustion, depression, and distress from this resident-generated material. (Appendix A) Each scenario scheduled for 15-minutes allowed between five and eight minutes for role-playing the scenario and up to 10 minutes for feedback and discussion. Prior to the role-play workshops, we had emailed participants three supplemental readings: the institution's resident physician resources, an article on promoting wellness in residency^1^ and an instructional handout on delivering feedback prepared by the author.

A total of 14 scheduled one-hour workshops were delivered, each including three residents and one faculty communication facilitator. Due to rotation commitments, some residents missed noon conferences, so the study team provided individual five-minute refreshers to encourage residents to add to the flipchart list of resident-identified burnout signs, symptoms, and precipitating situations factors.

The team also composed a fourth scenario using the additional signs, symptoms, and situations leading to burnout. The fourth scenario was included in the last five workshops. All scenarios consisted of situations, emotions, and behavioral manifestations of burnout previously identified by residents during workshops.

Since participating residents had previously learned to communicate empathy toward patients using the patient-centered interviewing technique NURS (i.e., Name, Understand, Respect and Support),^24^ this tool was used to encourage empathy toward each other during role-plays. Using NURS in this setting reinforced the use of this tool in patient care. Finally, the residents were provided laminated pocket cards listing the steps of the NURS communication tool. On the pocket card's flip side, the team listed free online and in-person assistive burnout recovery and wellness resources.

### Role-Playing Activities

During workshops, the three participating residents each adopted the role of a distressed colleague, helper colleague, and observer. The resident who role-played the distressed resident chose which of the three (i.e., later four) scenarios. Subsequently, the helper resident (who had not read the distressed resident Scenario), played the helper with matching scenario information provided. (See Appendix A for one example of scenario)

Neither the distressed resident nor the helper resident reviewed each other’s scenario. The observer resident observed intending to provide verbal and written feedback after the role-play. Each resident role-played a helper and distressed resident, and each was an observer/feedback giver.

Residents were then also given the option of creating their own role-play after providing a setup description for the helper. After role-playing, residents engaged in conversation about their feelings regarding their experience and personal reflections of their own or others' dysphoric feelings and experience supporting others or being supported by others. Personal examples of resident distress emerged spontaneously.

To create a safe learning environment, the author adhered to a structured self-awareness feedback format that emphasized positive and corrective communication skills. This feedback instructional document had been included in the supplementary reading emailed before the workshop. Specifically, the faculty facilitator asked the primary learner (i.e., the "helper") to reflect on what s/he did well, followed by the "distressed" colleague providing positive feedback regarding their conversation/intervention and the observer adding positive feedback. The faculty facilitator positively reinforced the effective strategies that residents used.

After positive feedback was provided using the same sequenced approach of the primary learner (helper resident) providing a self-critique, the "distressed resident" offering suggested improvements, and the observer offered helpful tips. Finally, the faculty facilitator instructed all resident participants concerning missed opportunities for empathy and support and improved communication strategies.

The faculty facilitator completed a resident discussion checklist consisting of qualitative and quantitative feedback, a narrative detailing concrete positive feedback and suggested improvements, and a quantitative rating scale documenting the presence or absence of adapted ACGME competencies of interpersonal skills and support/information giving, respectively. Discussion feedback checklists were immediately photocopied and distributed to residents for record-keeping, reference, and concrete feedback. (Appendix B).

### *Study* Measures

The team administered pre- and post-curriculum surveys that they had created, along with a post-curriculum reflection essay to evaluate their curricular aims. The five-item Likert scale pre-roleplaying burnout experience attitudinal questionnaire assessed residents' comfort, competence, confidence, knowledge, and atmosphere (Appendix C).

The study team’s 11-item Likert scale post-curriculum questionnaire was administrated immediately after the workshop to gauge residents’ satisfaction with the new curriculum, including usefulness (for practicing interpersonal and information-giving skills), feedback value, and teamwork/camaraderie, as well as perception of their overall experience (Appendix D). Likert scale questions data were analyzed as ordinal level, categorical data.

Data were analyzed using descriptive and unpaired inferential statistics with *IBM SPSS Version 25*. The analyst (NL in Acknowledgements) examined the frequencies and percentage responses of each question. Likert scale question responses were treated as ordinal-level, categorical data. The analyst used a chi-square analysis to see if there was a difference in proportion of favorable (i.e., Strongly Agree or Agree), neutral, or unfavorable (Disagree/Strongly Disagree) responses. The single-question reflection essay permitted residents to identify both personal and professional gains. (Appendix E)

Using a mixed-method design, the author’s team also used thematic content analysis with each member independently categorizing overall essay themes in participant comments. A specific member of the study team (DKT in Acknowledgments) reconciled differences. Finally, the faculty facilitator author shared personal observations with program leadership, providing additional insight into what worked well with the curriculum or could be improved in the future.

## RESULTS

All 42 (100%) of the eligible Internal Medicine (n = 36; 12 residents from each PGY 1, 2, & 3) and Transitional Year (n = six) residents opted to participate in a workshop and complete at least some pre-and post-curriculum survey items. When comparing pre- and post-training survey responses, statistically significant increases (*p* < .001) were found in overall resident-reported comfort, competence, confidence, and knowledge. Respondents also made positive comments regarding the “atmosphere” of the clinical teaching environment as a safe atmosphere in which to work and learn. (Table 2)

**Table 2.** Pre-Training ^a^ and Post-Training Comparisons of Resident Attitudes

	Strongly Agree/Agree		Neutral		Strongly Disagree/Disagree		P value
	Pre-training	Post-training	Pre-training	Post-training	Pre-training	Post-training	
**Comfort**	61% (*n* = 28/45)	97% (*n* = 40/42)	27% (*n* = 13/45)	3% (*n* = 2/42)	12% (*n* = 4/45)	0.0% (*n* = 0/42)	**< .001**
							
**Competence**	47% (*n* = 22/45)	95% (*n* = 39/41)	41% (*n* = 17/45)	5% (*n* = 2/41)	12% (*n* = 6/45)	0.0% (*n* = 0/41)	**< .001**
							
**Confidence**	73% (*n* = 35/45)	97% (*n* = 41/42)	22% (*n* = 9/45)	3% (*n* = 1/42)	5% (*n* = 1/45)	0.0% (*n* = 0/42)	**< .001**
							
**Knowledge**	33% (*n* = 14/45)	87% (*n* = 37/40)	46% (*n* = 21/45)	11% (*n* = 3/40)	21% (*n* = 10/45)	2% (*n* = 0/40)	**< .001**
							
**Atmosphere**	70% (*n* = 31/45)	98% (*n* = 42/42)	22% (*n* = 10/45)	0.0% (*n* = 0/42)	8% (*n* = 4/45)	2% (*n* = 0/42)	**< .001**

^a^ Three residents inadvertently took the pre-training survey twice. Due to survey anonymization, we were unable to remove these duplicate surveys.

There were no statistically significant response differences when comparing international medical graduates to American medical graduates or between residents' PGY training years.

### Thematic Analyses

The team’s thematic content analyses of reflection essays revealed 12 major themes. Six themes were incorporated in more than 25% of the residents' essays (Table 3). These represent six themes in which >25% of residents described in their personal essays. The expression of the remaining six themes not depicted in Table 3 ranged between 5% and 24%.

**Table 3.** Identified Themes in Residents’ Reflection Essays

Increased Knowledge/Skills: Communication Techniques	57%
Produced a Personal Commitment to Helping	41%
Increased Knowledge/Skills: Approach Strategies/Tactics	36%
Developed a Recognition of Importance of Subject Matter	29%
Increased Knowledge/Skills: Resources/Services Available	29%
Produced a Better Understanding of Oneself	26%

Resident participants most frequently cited the role-playing exercise as enhancing their knowledge base. The study team divided this into three subthemes, described in essays as knowledge of communication techniques (n = 24 (57.1%), knowledge of approach tactics or strategies (n = 15 (35.7%), and knowledge of residency resources (n = 12 (28.6%).

Narrative reflection exemplar comments include, "The way that I have been offering support may not have been the most optimal way, and today I learned about better techniques and methods to do that.;" "I became more confident in exploring my feelings and my concerns with my peers. I found out that we should all help each other, listen to each other, it was a great experience. I wish we have more sessions;" and "Everyone has different experiences, …… Even if you cannot completely relate, listening can help others open up and help them."

During workshops, some residents raised their personal life concerns (e g., a recent family death, personal illness, having suffered difficult situations listed in role-plays, etc.). For example, at the time of disclosure of distress at losing a close family member, the other residents immediately offered support, asking, "Man, why didn't you tell us?" Finally, approximately one year after workshops, three residents had anecdotally approached the faculty member to discuss their concerns or had sought mental health treatment resources.

## DISCUSSION

Our study results demonstrate that a peer-roleplaying curriculum methodology can help to improve residents’ communication skills and constructive relationships with colleagues to positively impact both clinical learning environments and individual residents’ well-being. Satisfying relationships have been shown in prior studies to build residents’ resilience levels and combat burnout.^12,25^ Earlier works have shown that the use of role-playing scenarios can influence well-being through: (1) naming/articulation of emotions; (2) relationships and enhanced social relatedness; (3) time for self-reflection; (4) permission to be vulnerable and ask for help; (5) building competency; and (6) self-care.^2,11,12,20^

Additionally, residents have the opportunity to observe fellow residents' distressed behavior before faculty or mentors, making them first-witnesses and often first-responders to distress.^26–28^ Finally, this type of educational approach can be used to address the stigma of discussing, requesting, and receiving assistance.^16,18,19^

During the current coronavirus pandemic, when many medical centers limit outside visitors, peer role-playing can be done safely with those already in "the bubble."^28^

Since residents' academic schedules dictated when they could participate in a workshop, new relationships among residents of various PGY levels were also ideally strengthened. Due to the personal and intimate nature of workshops, resident relationships may develop more effectively than during usual rotation assignments. The author has shown from their earlier study that residents' prior experiences participating in peer role-playing workshops on disclosing a medical error^29^ and using a familiar empathy tool (NURS)^24^ could create a more comfortable environment to discuss their burnout and dysphoric emotion experiences.

The fact that several residents later approached the faculty author to discuss concerns or had accessed mental health treatment resources may indicate that our curriculum could have been strengthened regarding how to establish a supportive relationship between faculty and residents. As the reflective essays and discussions demonstrated, utilizing peer role-playing supported increasing emotional intelligence, reading nonverbal cues, and paying attention to peers' well-being.

### Study Limitations

Since our study was limited to a single institution's Internal Medicine and Transitional Year residency programs with most international medical graduates, these results may not prove generalizable to other residency programs or institutions. Larger scale samples will be required to examine whether American and internationally trained residents might differ in their reports of this curriculum's usefulness.

### Curriculum Revisions

To address the larger scale problem of burnout in our residents, our team has since expanded this curriculum to include residents from each of our residency training programs. We also recommend combining all levels of residents so that junior residents and interns may benefit from the wisdom of senior residents.

## CONCLUSIONS

Our study team has concluded that this type of structured peer role-playing curriculum may offer a low-cost method to effectively destigmatize professional burnout and encourage residents to identify, approach and support distressed colleagues. Ideally, resident peers can develop their role as first-witnesses as sensitive, responsive peers for distressed colleagues.

### Conflict of Interest

None.

## Appendices

### APPENDIX A: Role-play Burnt-out and Depression Peer Support

#### Dr. B Burnt-out Resident

**Age:** 29 years old

**Location:** Clinic

**Problem:** You have been tardy to noon conference, made mistakes putting in orders, and neglected to give scripts to your clinic patients. Now you have been asked to talk to your mentor due to a complaint by a nurse. You feel like giving up, like you cannot do anything right. On top of this your husband/wife incurred medical bills that were not covered by insurance—now you are going to be more in debt. You are having trouble sleeping due to the anxiety all of this aroused. You want to be alone.

**Emotions:** You feel angry, scared, and hopeless, out-of-control over everything. You don’t know what to do.

#### Helper Colleague D

You notice that Dr. B has been late for rounds and lectures, which is unlike her/him. S/he is frowning, slumped in the chair making snide remarks about patients and colleagues. You overheard s/he being reprimanded by the senior for making mistakes.

Open the conversation.

### APPENDIX C: Pre-Roleplaying Experience Questionnaire

Internal Medicine ❑ PGY-1 ❑ PGY-2 ❑ PGY-3 ❑ PGY-4 ❑

Transitional Year ❑

Please mark your level of agreement with each of the following statements. Your sincerity or frankness is appreciated.

1. I am ***Comfortable*** in addressing difficult personal topics with my peers.
  ❑ Strongly Agree ❑ Agree ❑ Neutral ❑ Disagree ❑ Strongly Disagree
2. I feel ***Competent*** in communicating with my peers about such issues as depression or burnout.
  ❑ Strongly Agree ❑ Agree ❑ Neutral ❑ Disagree ❑ Strongly Disagree
3. I am ***Confident*** in offering support or assistance to my peers when discussing emotional problems related to depression or burnout.
  ❑ Strongly Agree ❑ Agree ❑ Neutral ❑ Disagree ❑ Strongly Disagree
4. I have a strong ***Knowledge*** base about available hospital resources for employees experiencing depression or burnout.
  ❑ Strongly Agree ❑ Agree ❑ Neutral ❑ Disagree ❑ Strongly Disagree
5. My training program provides residents with a ***'Safe' Atmosphere*** for learning how to offer support or assistance to their peers.
  ❑ Strongly Agree ❑ Agree ❑ Neutral ❑ Disagree ❑ Strongly Disagree

### APPENDIX D: Peer Roleplaying Curriculum Satisfaction Questionnaire

My participation in this training exercise...

1. Served to increase my comfort level in addressing difficult personal topics with my peers.
  ❑ Strongly Agree ❑ Agree ❑ Neutral ❑ Disagree ❑ Strongly Disagree
2. Helped to increase my competence in communicating with my peers about such issues as depression or burnout.
  ❑ Strongly Agree ❑ Agree ❑ Neutral ❑ Disagree ❑ Strongly Disagree
3. Was useful for practicing interpersonal skills.
  ❑ Strongly Agree ❑ Agree ❑ Neutral ❑ Disagree ❑ Strongly Disagree
4. Was useful for practicing information giving skills.
  ❑ Strongly Agree ❑ Agree ❑ Neutral ❑ Disagree ❑ Strongly Disagree
5. Increased my confidence in offering support or assistance to my peers when discussing emotional problems related to depression or burnout.
  ❑ Strongly Agree ❑ Agree ❑ Neutral ❑ Disagree ❑ Strongly Disagree
6. Provided immediate feedback that was valuable to me.
  ❑ Strongly Agree ❑ Agree ❑ Neutral ❑ Disagree ❑ Strongly Disagree
7. Created a 'safe' atmosphere for learning how to offer support or assistance to my peers.
  ❑ Strongly Agree ❑ Agree ❑ Neutral ❑ Disagree ❑ Strongly Disagree
8. Encouraged teamwork and camaraderie with my fellow residents.
  ❑ Strongly Agree ❑ Agree ❑ Neutral ❑ Disagree ❑ Strongly Disagree
9. Provided an opportunity to feel comfortable sharing personal experiences with colleagues.
  ❑ Strongly Agree ❑ Agree ❑ Neutral ❑ Disagree ❑ Strongly Disagree
10. Strengthened my knowledge base about available resources for employees experiencing depression or burnout.
  ❑ Strongly Agree ❑ Agree ❑ Neutral ❑ Disagree ❑ Strongly Disagree
11. My overall experience with today's role-playing session was:
  ❑ Very Good ❑ Good ❑ Neutral ❑ Not Good

### APPENDIX E: Peer Roleplaying Curriculum Reflection Essay

Regarding your own personal growth and development as a physician in such key areas as communication, respect, and sensitivity—would you please briefly describe a key lesson that you learned today.
